# The role of spontaneous and evoked neuronal activity in protection from impending stroke in rat model of permanent middle cerebral artery occlusion during the hyperacute state

**DOI:** 10.21203/rs.3.rs-5220780/v1

**Published:** 2025-05-06

**Authors:** Hayden C. Malone, Mehwish S. Bhatti, Ron D. Frostig

**Affiliations:** University of California, Irvine; University of California, Irvine; University of California, Irvine

## Abstract

Using an ischemic stroke rat model by applying middle cerebral artery occlusion (pMCAo), we have previously demonstrated that protection of the ischemic territory can be achieved by providing intermittent sensory stimulation within a period of two hours following the occlusion. Beyond this period, sensory stimulation becomes deleterious and results in infarct. We have further demonstrated that such sensory-based protection depends on the integrative role of activated synapses, activated neurons activated astrocytes, and activated blood vessels. By using the same rat pMCAo model for the current study, we hypothesized that all such activations are potentially triggered by sensory stimulation-based evoked neuronal activity within the ischemic cortex, that in turn triggers the various processes that lead to protection. To test this hypothesis, we used functional imaging and postmortem histology and selectively blocked spontaneous or evoked neuronal activity within the ischemic territory by local administration of lidocaine. Our findings demonstrate that the ischemic cortex is extremely sensitive, as clear functional blockage at the site of lidocaine diffusion and a corresponding infarct at the same location were found for both spontaneous activity and sensory-based evoked activity. Furthermore, the extreme sensitivity of the ischemic cortex is demonstrated by the detrimental effects of Phosphate Buffer Saline (PBS) application if protective sensory-based stimulation is not present following pMCAo. We conclude that neuronal activity, either spontaneous or evoked, within the ischemic cortex is pivotal for protection during the early hyperacute phase of ischemic stroke.

## Introduction

Historically in stroke research, the role of neuronal activity in deterioration to ischemic stroke or in protection from ischemic stroke has not been recognized as a significant factor. Regarding protection, our laboratory’s research has repeatedly demonstrated that protection from impending ischemic stroke during the clinically critical hyperacute phase of stroke depends on sensory (whisker) stimulation that activates the ischemic area. Previous findings from our laboratory have focused on *in-vivo* experiments in anesthetized and awake behaving rats during the hyperacute phase following dorsal permanent middle cerebral artery occlusion (pMCAo) demonstrating consistent results regarding protection vs. deterioration to infarct and their relationship to sensory stimulation (for our earlier review see ^1^, for more recent independent reviews see ^2,3^). Employing functional imaging (Intrinsic Signal Optical Imaging (ISOI)), flow imaging (Laser Speckle Imaging (LSI)), neuronal recordings, histology, and behavioral assessments, our first publication^4^ demonstrated that protection from impending ischemic stroke following dorsal pMCAo model (double ligation and transection at the M1 level^5^) is achievable at any time within the first 2h post pMCAo by delivering intermittent tactile stimulation of single whisker or the entire whisker array. Beyond the 2h protection period, this treatment is no longer protective, resulting in infarct formation ^4^. Sensory evoked retrograde blood flow within the permanently occluded MCA is critical for this protection. Such retrograde flow originates from opening of distal pial collaterals with other cortical arteries (ACA, PCA) resulting in reperfusion of the ischemic MCA territory^4–6^. During the 2h treatment window, we found that recovery of a whisker functional representation in barrel cortex to pre-pMCAo baseline level was surprisingly similar in its temporal dynamics regardless of time that the protective treatment starts: either immediately (+ 0h), 1h (+ 1h) or 2h (+ 2h) following pMCAo^6^ indicating that the cortex can self-protect even without sensory stimulation for 2h following pMCAo. Between 2h and 3h post-pMCAo there is a dramatic reversal, namely the same sensory stimulation results in infarct that is even larger than the one obtained in no-stimulation controls. By 5h post pMCAo the stimulation seems to no longer have an effect. Notably, this sensory-based treatment is also protective for 21–23 months old rats^7^ indicating a considerable age-range effectiveness – an important finding considering the association of age and stroke in humans^8^. We have also demonstrated that this treatment is independent of anesthesia type^9^, and that the same protection was achieved in behaving rats that self-stimulate their sensory-motor systems while exploring an enriched environment, even after their whiskers were removed^10^ therefore demonstrating that it is a general sensory-motor activation of the ischemic MCA territory that is necessary for early protection. We also found that protection is long term: even 4 months post pMCAo rats remain protected ^11^. The same treatment however does not seem to protect popular strains of mice^12^, or spontaneously hypertensive rats^13^. Finally, we demonstrated that following pMCAo collateral flow is surprisingly dynamic in its spatiotemporal flow patterns and that protective sensory stimulation always enhances its velocity and flux although they remain relatively weak ^14^. Recently, using detailed, widespread and continuous neuronal recordings before and 5h after pMCAo from the entire depth the ischemic area by applying a 32 microelectrodes array, we have discovered that immediately (within 5 minutes) following pMCAo there is a widespread, strong increase in synchronicity of spontaneous (but not evoked) local field potentials (LFPs) that is predictive of pMCAo outcome and is desynchronized by sensory stimulation treatment within the first 2h after pMCAo, but not at 3h after pMCAo ^15,16^. Finally, we have demonstrated that astrocytes have an important role in sensory-based protection by supplying lactate to the neurons in the ischemic area using the astrocytes-to-neurons shuttle (ANLS) ^17^. Notably, our findings have followed an earlier study suggesting a protective role of sensory stimulation^18^ and have also been independently replicated in several other labs all demonstrating protection following early sensory treatment ^19–22^. Further evidence supporting the importance of such protective activation was provided by the protective effects of transcranial electrical stimulation^23–25^, transcranial focused ultrasound stimulation^26^, and optogenetic stimulation of the ischemic MCA territory in rodents ^27^.

Taken together, the above results strongly suggested that sensory stimulation-based protection depends on direct activation of neurons and astrocytes in the ischemic area during the hyperacute state. However, this hypothesis has never been directly tested. Applying functional imaging, pharmacological manipulations and post-mortem TTC staining of the cortex, our aim was to directly demonstrate that both spontaneous neuronal activity and evoked neuronal activity have each a role as a trigger for the protection process. Here we demonstrate that pharmacologically blocking either spontaneous or evoked neuronal activity after pMCAo results in deterioration to stroke and therefore directly establishing the role of neuronal activity in protection during the hyperacute state following pMCAo, findings that are consistent even for two different types of sensory stimulation.

## Materials and Methods

All conducted procedures strictly followed the National Institute of Health (NIH) guidelines and were approved by University of California Irvine Animal Care and Use Committee (IACUC, protocol: AUP-21–065). All methods reported are in accordance with the ARRIVE guidelines. All the surgical procedures, data acquisition and analysis methodology follow our previously published methods ^17^ and are briefly described here.

### Animals

Forty male Sprague Dawley (SD) rats, 230–450g (Charles River Laboratories, Wilmington, MA, USA) were individually housed in enriched cages placed in a temperature, humidity and light-controlled room (twelve-hour-cycle: 6am-6pm). Each rat was handled daily for twenty minutes for 3–5 days until the day of experiment.

### Reagents

Lidocaine is a potent sodium channel blocker broadly used in a wide range of preparations for pain management and local anesthetics. Long-term exposure and high concentrations of lidocaine can be potentially lethal to the neural tissue, therefore preliminary work was carried out to find out the optimal dosage that bears no harmful effect on the local neural-circuitry response to C2-WFR (whisker functional representation of C2 whisker stimulation) in healthy rats. 5% lidocaine was used with sterilized phosphate buffered saline (PBS, pH = 7.4). 2,3,5-triphenyltetrazolium chloride (TTC) is used for post-mortem histology to highlight and quantify the infract area. All drugs are purchased from Sigma Aldrich, Saint louis, MO, USA.

### Experimental Design

**Figure 1:**
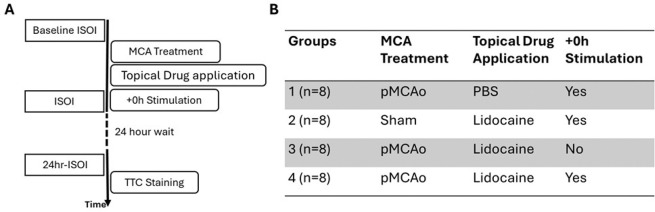
Experiment Timeline and Group Description. A) shows the detailed timeline of experiments B) shows the four groups with MCA, topical drug and the stimulation treatment delivered for each experiment group. ISOI refers to Intrinsic Signal Optical Imaging. +0h refers to 5 Hz whisker stimulation applied right after the onset of permanent Middle Cerebral Artery occlusion (pMCAo). B)shows the details of interventions, namely MCA, whisker stimulation and drug treatment that each group of rats received. Rats in group 1 (n=8) received permanent Middle Cerebral Artery Occlusion (pMCAo), application of vehicle (PBS) and immediate post occlusion whisker stimulation (+0h). Rats in group 2 (n=8) received sham treatment for MCA occlusion, 5 % lidocaine and +0h stimulation. Rats in group 3 (n=8) received pMCAo, 5 % lidocaine but no whisker stimulation. Rats in group 4 (n=8) received pMCAo, 5 % lidocaine and +0h whisker stimulation. Groups 1–3 serve as three different control groups to group 4.

In this study, we applied a within subject design where a baseline is established and compared to 24-hour post manipulation result. Figure 1A shows the timeline of all experiments. Forty SD rats were randomly assigned to one of the five experimental groups by an experimenter, blind to the experiment protocols as shown in Fig. 1B.

### Presurgical preparations

Rats were briefly anesthetized with 4% Isoflurane at the start of the experiment, weighed and injected intraperitoneally (i.p.) with sodium pentobarbital bolus (55 mg/kg, body weight (bw)). Supplemental injections (14 mg/kg bw) were given as necessary to maintain loss of withdrawal reflex to toe/tail pinch. C2 whisker was marked, and the remaining whiskers were cut before head-fixing the rat in stereotaxic restraint. At the beginning and end of day 1 of the experiment 5% warmed dextrose (3mL, subcutaneous) and atropine (0.05 mg/kg, bw, intramuscular) were administered. Rectal probe is used to continuously monitor the body temperature which is maintained at 37° Celsius by a self-regulating thermal blanket. Heart rate and partial oxygen saturation were monitored (Kent Scientific Mouse Stat Jr.) throughout the experiment.

### Surgical preparations

The surgical area is cleaned, and local anesthesia (2% lidocaine) is applied subcutaneously. After the midline incision was made, the soft tissue underneath was resected to expose a ~ 7 mm × 7 mm ‘imaging’ area of the skull over the left primary somatosensory cortex (rostro medial corner positioned caudal and lateral from bregma) was thinned to ~ 24–32 μm using a dental drill. Baseline C2 whisker functional representation (WFR) using intrinsic signal optical imaging (ISOI) was obtained for two types of whisker stimulation protocols (see details below in the imaging/whisking protocol).

### Skull-dura slits

The procedure of dura slits was adapted and improved from our previously reported procedure ^17^. In this procedure full craniotomy of the imaging window was replaced by small, aligned skull slits with dura slits for drug diffusion. The slit of desired size was made in the dura with the sharp part of the 30G needle by lifting it gently under 40x magnification via surgical microscope. Care is taken to make no contact with the underlying neural tissue.

### Permanent dorsal middle cerebral artery occlusion (pMCAo)

This procedure was first demonstrated and described by Davis et al ^5^. Briefly, the base of the left middle cerebral artery is permanently occluded at the M1 segment blocking flow to all MCA cortical branches. To do this, the skull and dura are carefully removed from a 1.5 × 1.5 mm ‘surgical window’ just anterior and lateral to the imaging window (over the M1 segment of MCA, just distal to MCA’s lenticulostriate branches and proximal to any cortical branching). A small needle is threaded with 8–0 silk thread and passed through the pial layer of the meninges, below MCA. The two parts of threads are separated and then secured tightly around the MCA. Care is taken to avoid hemorrhaging the artery and nearby vessels. In some cases, a terminating small branch of a vein that is located very close to MCA was also unavoidably occluded, but the results in these cases were not different from only MCA occlusion cases. The experiments are terminated if MCA or any other major vessel in the vicinity is hemorrhaged. For the sham surgery group, the same procedure is used but without tying the artery.

### Drug Administration

A petroleum jelly (Vaseline) well was made around the imaging window and filled with saline for baseline ISOI. The Vaseline well is filled with drug/vehicle according to the rat-assigned group for 30 minutes after pMCAo within the critical time window for sensory-based neuroprotection.

### Postsurgical preparations

Analgesic (Flunixin meglumine) was injected subcutaneously (2 mg/kg) at the end of the imaging sessions on day 1. The closed wound was covered with topical antibiotic (Animax, Petco) and 2% agarose. Rats were monitored while recovering from anesthesia on a heating blanket (Adroit Medical Systems). Rats were then returned to a clean cage to recover overnight prior to 24 hours (referred to as 24hr) ISOI. After 24hr ISOI, rats were euthanized, with lethal dose of Euthasol (2 ml, ip) and prepared for histology.

### Intrinsic signal optical imaging (ISOI)

The details of ISOI data acquisition and analysis can be found in our previous work^28–30^. Briefly, a charge coupled device (CCD) camera (16-bit Cascade 512, Photometrics, Tucson, AZ, USA) or a scientific complementary metal-oxide-semiconductor (sCMOS Prime 95B, Photometrics, Tucson, AZ, USA), both equipped with an inverted 50 mm AF Nikon lens (1:1:8, Melville, NY, USA) combined with an extender (model PK-13, Nikon, Melville, NY, USA) is used for imaging and controlled by V + + Precision Digital Imaging System software (Digital Optics, Auckland, NZ). Data is acquired in 100 ms frames that are summed to 500 ms frames to improve signal-to-noise ratio. The cortex is illuminated with a single red-light emitting diode (635 ± 15 nm wavelength).

### Imaging/whisking protocols

Two types of whisker stimulation protocols, sparse and condensed, were applied during imaging at baseline and 24 hours. Both protocols were also applied in our previous work^17^.

#### Sparse protocol:

During each 15 s trial of sparse protocol, 1.5 s of pre-stimulus data, 1 s of during stimulus and 13.5 s of post-stimulus data was collected, with a 6 ± 5 sec random inter-trial interval. Sparse protocol was used as it produces the same temporal sequence of functional response phases as also imaged by powerful (high-Tesla) BOLD-fMRI, a protocol that was consistently applied in all our previous work.

#### Condensed protocol:

During each 4.5 s trial of condensed protocol, 1.5 s of pre-stimulus data, 1 s of during stimulus and 2 s of post-stimulus data was collected, with a 1 sec constant inter-trial interval and 1 sec for data writing to hard drive. A condensed protocol was used because it mimics the naturalistic pattern of whisking in awake, behaving rats.

For both protocols, stimulus consisted of a single whisker being deflected by 9° in the rostral-caudal direction at a rate of 5 Hz for a total stimulus duration of 1 second in each trial. Data is collected in blocks of 64 stimulation trials for sparse and in blocks of 40 for condensed whisking protocol. All post pMCAo (+ 0h stimulations) of 100 condensed whisking stimulation. In all animals, 100 trials of condensed whisker stimulation immediately after pMCAo are referred to as + 0h stimulation.

### Imaging analysis

**Figure 2:**
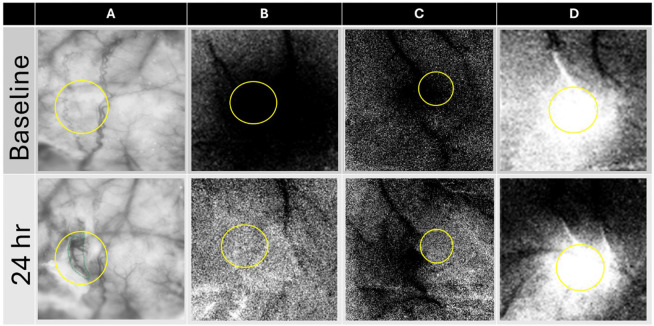
Selection of region of interest for spatial analysis. A) shows region of interest selected based on the landmarks of the surface vasculature of the imaging window. Same region is selected for baseline and 24 hours/24hr after the pMCAo for areal quantification. The region is selected (in yellow) around the drug slit (in green) for quantification of changes post intervention. The drug slits were of different sizes and orientation cut right above the region of activity and therefore the region of interest (ROI) drown around it is of different sizes/shapes. B) shows ROI), selected for a representative case where no WFR recovery of the initial dip for condensed protocol is observed after 24 hours. C) shows ROI, selected for a representative case where only partial WFR recovery of the initial dip is observed after 24 hours. The partial recovery is however only outside the region of interest. D) shows ROI, selected for a representative case where full WFR recovery of the overshoot for sparse protocol is observed after 24 hours. The comparison is only made inside the selected region of interest for baseline and 24 hours post pMCAo. The images are thresholded at ±2.5 × 10^−4^.

**Figure 3:**
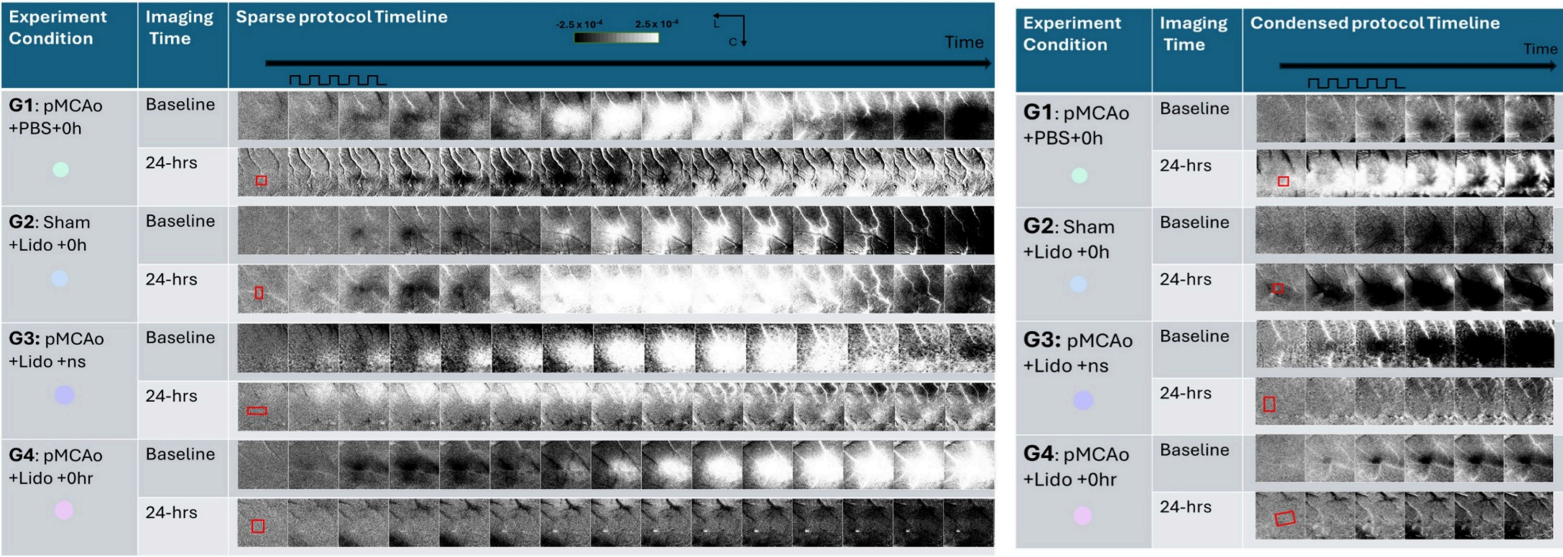
Representative result of ISOI from each group using sparse whisker stimulation protocol (top) and condensed whisker stimulation protocol (bottom). Baseline result on top and 24hr result below for direct comparison. Red boxes in peristimulus frame (first frame) indicate location of skull-dura slit for drug diffusion. The pulse train indicates start and finish of 1 second 5Hz- whisker stimulation. Linear gray scale bar indicates intrinsic signal strength ± 0.00025, C and L denotes caudal and lateral. Area encompassed by imaging is 7×7mm. Frame duration =500 ms. White and black streaks are vessel artifacts.

From raw images, ratio images were created from calculating fractional change (FC) values by dividing each 500 ms frame of post-stimulus signal activity by the 500 ms frame of pre-stimulus intrinsic signal activity collected immediately before stimulus onset. WFR for sparse protocol shows three distinct phases following stimulation. The three phases are, in the order of their appearance, the initial dip (typically dark in relation to pre-stimulus baseline), the overshoot (typically bright in relation to baseline) and the undershoot (topically dark in relation to baseline)^30^. When the cortex is illuminated by red light (635 nm), the initial dip represents the increase in deoxyhemoglobin due to immediate oxygen support to neuronal activation, the overshoot represents the vascular response of blood flow into the activated area due to increase in oxyhemoglobin (like the BOLD response of fMRI). As in our previous publications, we have analyzed only the first two phases and therefore Fig. 3 in the Results section shows only the initial 7s of data acquisition. The condensed protocol, however, only shows a single phase of growing initial dip. For spatial analysis a region of interest is carefully selected based on the landmarks of surface vasculature in the imaging window overlayed on the selected ratio image used for quantification as shown in Fig. 2.

#### Spatial Analysis – *Sparse protocol*:

The ratio images containing the maximum areal extent for each intrinsic signal phase were selected and Gaussian filtered (half width = 5). The areal extent was quantified at a threshold level of 1.75 × 10^− 4^ for initial dip and at 3.5 × 10^− 4^ (fractional change ΔR/R units) threshold for the overshoot, away from zero. Peak amplitude was quantified in fractional change units for the pixel with peak activity within the areal extent. Notably, comparing changes between baseline and 24 hours in all experimental groups, the pixel of peak intensity was selected within the area of the skull-dura slit (drug diffusion area).

#### Spatial Analysis – *Condensed protocol*:

The ratio images 0.5–1.0 second after the stimulus delivery was selected and Gaussian filtered (half width = 5). The areal extent was quantified at a threshold level of 2.5 × 10^− 4^ (fractional change ΔR/R units) threshold, away from zero. Peak amplitude is calculated in a region of interest inside the drug diffusion area for the same pixel at baseline and 24hr after pMCAo.

### Histology (staining for infarct)

The brain was sectioned into 2 mm coronal slices and incubated in 2% TTC solution at 37°C for 20 min in the dark^31^. TTC is enzymatically reduced, producing formazan (a bright red byproduct), by dehydrogenases in active mitochondria. Red stain intensity correlates with the number and functional activity of mitochondria, unstained (white) areas are indicative of infarct^32^.

### TTC analysis

The TTC-stained sections were photographed with a digital camera, and images were analyzed using ImageJ software (National Institute of Health) ^33^. Individual slice infarct volumes were calculated by multiplying the infarct area of each slice by the slice thickness, and total infarct volume was determined by summation of volumes across slices. Final volumes were then corrected for edema by normalizing the volume of the ischemic hemisphere to the contralateral hemisphere. An observer blind to the experimental groups performed the volume calculation. Small damage at the pMCAo surgical site was readily distinguished from the large ischemic infarct and was excluded from infarct analysis. The images with infarct in groups 3–5 were superimposed on the images obtained from the rat brain atlas (Paxinos and Watson)^34^. The atlas images were selected based on landmarks observed in the slices.

### Statistical analysis

For ISOI-WFR imaging data, repeated measures analysis of variance (RM-ANOVA) was performed to analyze potential differences between baseline and 24 hours after pMCAo in all experimental groups. Repeated measures were performed for one between subject’s variable (experiment groups, 1–5) and one within subjects (time, baseline vs 24h) followed by post hoc contrasts to identify which groups differed from baseline at 24-hours. Alpha level was set to 0.05, and Bonferroni/Sidak corrections were applied to account for multiple contrasts. Infarct volume comparisons were performed by employing two-sample t-tests. One-way ANOVA was performed with post hoc Bonferroni corrections to ensure that no statistical difference exists between baseline values of all groups (1–5). One-way ANOVA was performed for comparison of infarct volume obtained from analysis of TTC analysis through ImageJ ^33^. All statistics and plotting were performed using PRISM (GraphPad version 9). Results are expressed as means and standard errors.

## Results

### Suppressing neuronal activity after ischemic onset prevents recovery

Figure 3 includes a representative result for all experimental groups of whisker functional representation (WFR) gained from sparse and condensed protocols. For both sparse (top half) and condensed (bottom half) protocols, suppressing neuronal activation with lidocaine during the treatment period post pMCAo leads to affected neurons’ inability to recover functionally as seen in groups 3 (pMCAo + lid + ns) and 4 (pMCAo + lid + 0hr). At 24-hr assessment, condensed and sparse imaging protocols reveal that the WFR is reliably absent from the area of drug diffusion. This data demonstrates that blocked neurons were unable to recover their functional representation in areas corresponding to the diffusion of lidocaine, indicating a sustained functional impairment. Group 5 (pMCAo + PBS + ns) showed similar 24-hr functional impairment due to the absence of neuronal activity through no-stimulation. Conversely, Groups 1 and 2 (vehicle or sham surgery, respectively) baseline vs. 24-hr WFR data remained consistent, demonstrating complete recovery at 24-hr. The stability of these 2 groups suggests that without lidocaine intervention, neural mechanisms can still take place under the stress of pMCAo to provide neuroprotection. The stark contrast between all the experimental groups strongly supports the idea that suppressing neuronal activation with lidocaine disrupts functional recovery mechanisms that would otherwise be observed following ischemic insult. Supplementary Fig. 1 demonstrates that lidocaine completely blocks evoked cortical activity during day 1 of the experiment following sham surgery.

### Spatial quantification of WFR response for each experimental group at baseline and 24hrs after pMCAo

**Figure 4:**
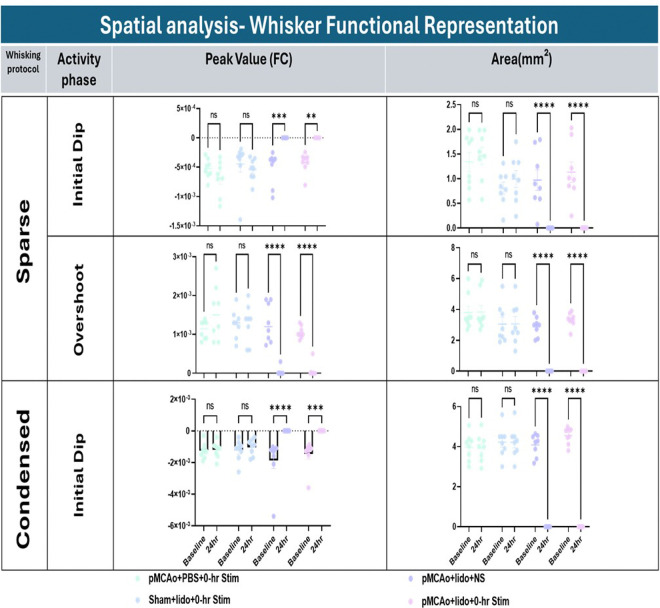
Spatial quantification of ISOI-WFR using sparse and condensed protocols. Top: Initial dip peak amplitude and area from sparse whisker stimulation at baseline vs. 24hr for each group. Significant differences found in groups 3 and 4 baseline vs. 24hr for both peak area and amplitude. Middle: Overshoot peak amplitude and area from sparse whisker stimulation at baseline vs. 24hr for each group. Significant differences found in groups 3 and 4 baseline vs. 24hr for both peak area and amplitude. Bottom: Initial dip peak amplitude and area from condensed whisker stimulation at baseline vs. 24hr for each group. Significant differences found in groups 3 and 4 baseline vs. 24hr for both peak area and amplitude.

Figure 4 presents quantification of areal extent (Area) and Peak Amplitude (or value) results from initial dip and overshoot phases of WFR using sparse and condensed stimulation protocols. Quantification was performed on the frame that shows the maximum areal extent. The results from the sparse protocol are detailed in the top and middle sections of the figure. Groups 3–5 exhibited significant reductions in both variables at 24h assessment at both initial dip and overshoot phases. These findings indicate suppressing neuronal activity in the presence of ischemia halts the cortex ability for functional recovery within 24 hours after pMCAo.

In contrast, no significant effects were observed in area or peak amplitude from the vehicle or sham surgery groups. The Condensed protocol yields similar results, where significance is seen only when both stroke and neuron inhibition is present (groups 3–5). Again, group 1 and 2 had no significant changes at 24 hrs post treatment. This suggests that in the absence of neuronal inhibition, functional recovery is not hindered, even following an ischemic event.

### Inhibition of neuronal activity shows corresponding cortical infarct

**Figure 5:**
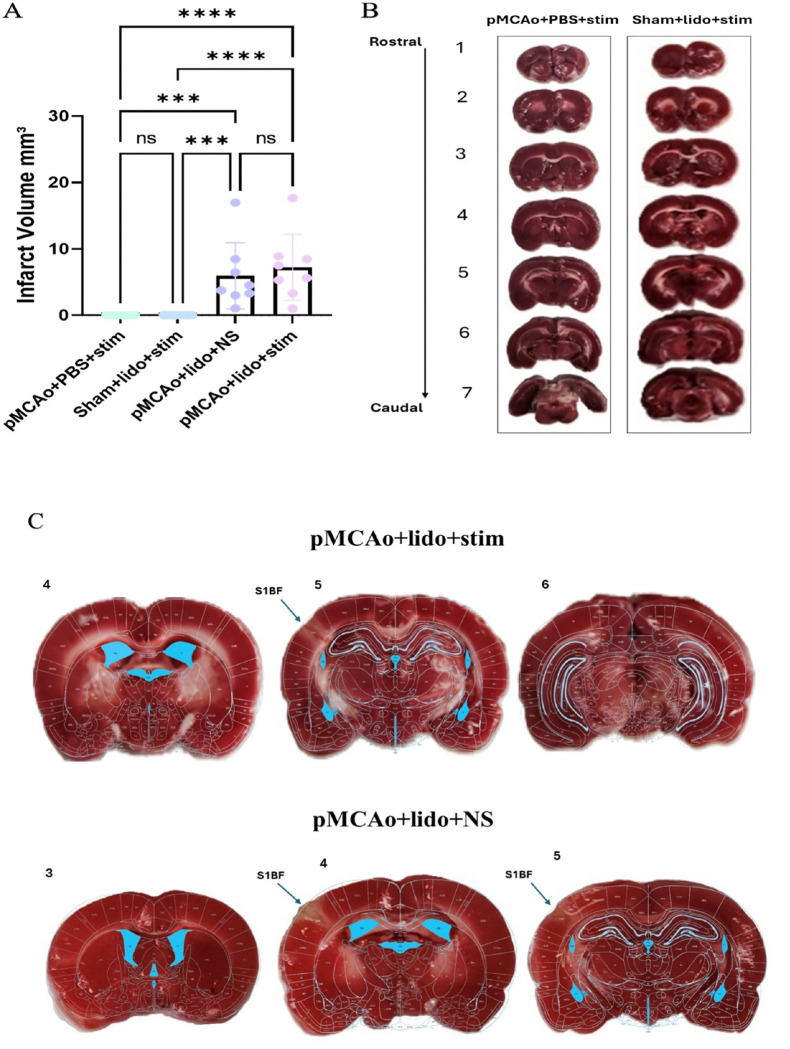
Infarct quantification. Infarct quantification for all experimental groups, providing insight into structural damage and localization following different interventions. (A) Infarct volume of all groups (1–4 ****p<0.0001, ***p<0.0002). Group 3 and 4 show marked increases in infarct volume compared to groups 1 and 2, which suffer no infarct. (B) Representative examples of TTC-staining confirm absence of infarct or structural damage in groups 1 or 2. These images confirm that without the presence of both lidocaine and occlusion, cortical structures remain intact. (C) Representative case for TTC stained brain from group 3 and 4, focusing on slices before the infarct begins, where the diffusion site was opened allowing for infarct, and where the infarct ends. Overlaying cortical map from Paxinos and Watson rat brain atlas reveals infarct is specific and contained to primary somatosensory barrel field (S1BF).

Infarct volume was calculated for all experimental groups, illustrated in Fig. 5A. Representative cases of Groups 1 and 2, which suffered no observable damage to structure, as confirmed by absence of infarcted tissue, are depicted in Fig. 5B. These results are consistent with the lack of functional impairment observed in the imaging data for these groups, reinforcing the importance of neuron stimulation under the stress of ischemic factors to achieve neuroprotection.

In contrast, infarcts were observed in representative cases of groups 3–5 as shown in Fig. 5C. Figure 5C shows representative cases and Paxinos and Watson’s rat brain atlas was overlaid onto representative slices from groups 3–5, clearly localizing the infarct to the primary somatosensory barrel field (S1BF) region. This region corresponds precisely to the area of lidocaine diffusion, and in turn, where neurons were prevented from neuronal activation either in response to whisker stimulation or spontaneous activity for both groups. Two representative cases are provided for group 5, which displayed a different distribution of infarct pattern (sometimes localized to S1BF, other times sustaining a larger infarcted area overreaching the bounds of S1BF).

The confinement of the infarct to the S1BF in these groups highlight the direct relationship between the region of drug diffusion, and the population of neurons affected by lidocaine resulting in their inability to recover. These results suggest that the suppression of neuronal activity, in tandem with ischemia, leads to structural damage as observed from the infarcted tissue in groups 3–5.

### Correlation of infarct volume and area of drug slit

**Figure 6:**
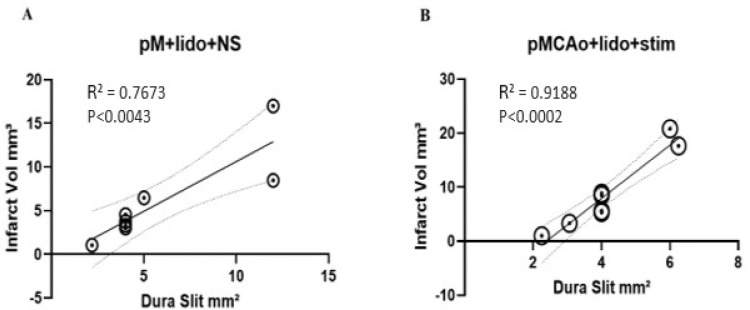
Linear regression of slit size and infarct volume from 8 animals of groups 3 and 4, note that the x axis on different scales for the groups due to slit size variations. (A) Group 3 displays a significant linear correlation with total slit size and infarct. (B) Group 4 displays extremely significant linear correlation with total slit size and infarct. As slit is made larger, larger infarcts are inflicted. Bold circles represent overlap of more than one case.

The infarct area consistently encompassed the entire region of drug diffusion. Furthermore, Fig. 6 shows that in groups 3 and 4 the size of the dura slit opening made for drug diffusion was highly predictive of total infarct volume as confirmed by significant linear regressions. Generally, the larger the drug slit, the larger the population of neurons were inhibited, leading to greater infarcts. This finding suggests the extent of area exposed to lidocaine during stroke evolution directly influences the degree of structural damage for both groups 3 and 4 with extremely strong correlation.

## Discussion

Our previous results regarding sensory stimulation-based protection from ischemic stroke have revealed so far three underlying mechanisms involving active neurons, active astrocytes, and blood vessels that support such protection. They include (1) sensory stimulation-based activation of collaterals’ blood flow originating from other cortical arteries resulting in a new source of retrograde blood supply within the occluded MCA that consequently results in reperfusion of the ischemic area,^4^ (2) sensory stimulation-based desynchronization of a strong and widespread buildup of spatiotemporal synchronization of spontaneous LFP in the ischemic territory following pMCAo^15,16^, and (3) sensory stimulation-based activation of the astrocytes to neurons lactate shuttle (ANLS) supporting neuronal activity within the ischemic area ^17^. Since sensory stimulation typically results in an increase in neuronal activation in the ischemic area and consequently increase in astrocytes activation ^35^, it led to the hypothesis that such increase triggers all the underlying mechanisms of protection, as non-stimulus controls in all three cases didn’t exhibit any of the effects described above. To test the hypothesis regarding the importance of neuronal activity in triggering protection, the current study used the sodium channel blocker lidocaine to block neuronal activity in the ischemic cortex during the clinically important hyperacute phase of ischemic stroke.

Using functional imaging (ISOI) before pMCAo (baseline) and 24h following pMCAo, and using post-mortem TTC histology in the same rats, results showed strong support for our hypothesis. The two lidocaine-treated groups following pMCAo showed lack of functional activation following sensory (whisker) stimulation at ischemic area treated with lidocaine, findings that were further supported by the corresponding TTC histological findings that showed infarcted tissue at the lidocaine treated area. Notably, these functional imaging and histological results were consistent despite the administration of two very different types of sensory stimulation, i.e., condensed vs. sparse, and therefore suggest that these findings could be generalizable beyond the exact details of the stimulation parameters.

The results of blocking spontaneous activity seem surprising at first glance. Spontaneous activity is typically known to be weaker than evoked activity and therefore seem less expected to serve as a trigger of protection. However, previous results in mice using voltage sensitive dyes imaging in barrel cortex during spontaneous activity showed repeated activation of the cortical tissue by depolarizing waves ^36^. Such spontaneous activity could potentially explain our previous results that showed that the ischemic cortex can be protected for 2h following pMCAo without sensory stimulation, suggesting therefore that spontaneous activity can keep the cortex protected for a limited time despite being weaker than sensory evoked activity. But beyond the 2h protection window by spontaneous activity, the cortex deteriorates to ischemic stroke as judged by functional imaging and postmortem histology 24h after pMCAo, whereas administrating sensory stimulation following any time within the 2h period following pMCAo results in protection as judged 24h after pMCAo ^4,6^. Higher levels of spontaneous activity in the ischemic cortex in some rats could also potentially explain some cases that we have encountered where rats were found to be protected despite receiving pMCAo.

Another unexpected indication for the extreme sensitivity of the ischemic cortex are the findings from experimental group 5 (pMCAo + PBS + ns). It clearly shows that the application of PBS (Phosphate Buffered Saline) to the ischemic cortex is detrimental following pMCAo, specifically in the absence of stimulation, resulting in functional impairment and structural damage. PBS, widely used in many biological applications, is isotonic, pH neutral, buffered and nontoxic and therefore is an essential tool for maintaining physiological conditions and supporting cell viability. It seems, however, that for the extremely sensitive ischemic cortex the PBS molecular composition that is so essential for its successful applications, can be toxic to cortical activity. Group 5 results also add strong support to the importance of protection of the ischemic cortex by sensory-based activation, as other experimental groups containing PBS with the combination of sensory stimulation show functional and structural protection 24 hrs following pMCAo. It remains unclear why, compared to other experimental groups, group 5 shows an almost binomial spread of functional and structural results, and more research is needed to uncover the reasons.

Selecting a lidocaine concentration that would properly inhibit all neurons in the diffusion area, without inducing cytotoxicity was important. We conducted preliminary experiments to confirm that application of 5% lidocaine for half an hour does not influence the whisker functional response reassessed at 24hr. As in our previous publication^17^, we show here that the use of the dura slit method enables us to restrict drug diffusion in the cortex and consequently control the size of the resulting infarct, even when different opening sizes for drug delivery were used Fig. 6. The fact that in both studies different drugs were used (here lidocaine and previously 4-CIN (alpha-cyano-4-hydroxycinnamte)) suggests that the strong correlation between the area of the dura slit and the infarct volume could be holding irrespective of the drug used. Further research with different drugs is needed to further confirm this valuable finding, as controlling the volume of the infarct is an important tool for studying the effects of the infarct volume on various outcomes of deterioration of the ischemic cortex to infarct.

The success of employing the lidocaine in this study raises another potential hypothesis. We have found in the past that when the protective stimulation is administered 3–5h after pMCAo, it is no longer protective but instead creates an infarct that is even larger than the one created with no stimulation as assessed by imaging and histology 24h after pMCAo ^4,6^. Future research will be conducted to test the hypothesis that the administration of a blocking agent such as lidocaine specifically during the 3–5h period after pMCAo, could also protect the cortex from deterioration to infarct. If so, both protection and deterioration to infarct during the hyperacute phase would critically depend on the appropriate modulation of neuronal activity.

Notably, the successful implementation of non-invasive techniques to enhance or inhibit cortical activity for rehabilitation after stroke seem to generalize the positive effects of cortical activity modulation even beyond the hyperacute state (reviewed ^37^) and therefore highlights a growing understanding regarding the significant role of neuronal activity in protection, deterioration to infarct, and rehabilitation after stroke.

## Conclusion

Our results support the hypothesis that both spontaneous activity and sensory-based evoked activity during the hyperacute state following pMCAo activate the cortical ischemic tissue, activation that serves as a potential trigger for several underlying mechanisms that result in protection of the cortical tissue from impending ischemic stroke.

## Data Availability

The dataset generated from raw Intrinsic signal optical images is available on request from the corresponding author.

## References

[1] Frostig R. D., Lay C. C. & Davis M. F. A rat’s whiskers point the way toward a novel stimulus-dependent, protective stroke therapy. Neuroscientist 19, 313–328, doi:10.1177/1073858412462607 (2013). 23047156 PMC3710106

[2] Baron J. C. Protecting the ischaemic penumbra as an adjunct to thrombectomy for acute stroke. Nat Rev Neurol 14, 325–337, doi:10.1038/s41582-018-0002-2 (2018). 29674752

[3] von Bornstadt D. Sensory stimulation in acute stroke therapy. J Cereb Blood Flow Metab 38, 1682–1689, doi:10.1177/0271678X18791073 (2018). 30073883 PMC6168904

[4] Lay C. C., Davis M. F., Chen-Bee C. H. & Frostig R. D. Mild sensory stimulation completely protects the adult rodent cortex from ischemic stroke. PLoS One 5, e11270, doi:10.1371/journal.pone.0011270 (2010). 20585659 PMC2890583

[5] Davis M. F., Lay C. & Frostig R. D. Permanent cerebral vessel occlusion via double ligature and transection. J Vis Exp, doi:10.3791/50418 (2013). PMC3845834 23912746

[6] Ma H. Thrombolysis Guided by Perfusion Imaging up to 9 Hours after Onset of Stroke. N Engl J Med 380, 1795–1803, doi:10.1056/NEJMoa1813046 (2019). 31067369

[7] Lay C. C., Davis M. F., Chen-Bee C. H. & Frostig R. D. Mild sensory stimulation reestablishes cortical function during the acute phase of ischemia. J Neurosci 31, 11495–11504, doi:10.1523/JNEUROSCI.1741-11.2011 (2011). 21832179 PMC3162364

[8] Lay C. C., Davis M. F., Chen-Bee C. H. & Frostig R. D. Mild sensory stimulation protects the aged rodent from cortical ischemic stroke after permanent middle cerebral artery occlusion. J Am Heart Assoc 1, e001255, doi:10.1161/JAHA.112.001255 (2012). 23130160 PMC3487352

[9] Ovbiagele B. & Nguyen-Huynh M. N. Stroke epidemiology: advancing our understanding of disease mechanism and therapy. Neurotherapeutics 8, 319–329, doi:10.1007/s13311-011-0053-1 (2011). 21691873 PMC3250269

[10] Lay C. C., Jacobs N., Hancock A. M., Zhou Y. & Frostig R. D. Early stimulation treatment provides complete sensory-induced protection from ischemic stroke under isoflurane anesthesia. Eur J Neurosci 38, 2445–2452, doi:10.1111/ejn.12217 (2013). 23586641 PMC3735829

[11] Lay C. C. & Frostig R. D. Complete protection from impending stroke following permanent middle cerebral artery occlusion in awake, behaving rats. Eur J Neurosci 40, 3413–3421, doi:10.1111/ejn.12723 (2014). 25216240 PMC4218886

[12] Hancock A. M., Lay C. C., Davis M. F. & Frostig R. D. Sensory Stimulation-Based Complete Protection from Ischemic Stroke Remains Stable at 4 Months Post-Occlusion of MCA. J Neurol Disord 1, 135, doi:10.4172/2329-6895.1000135 (2013). 24634892 PMC3952275

[13] Hancock A. M. & Frostig R. D. Testing the effects of sensory stimulation as a collateral-based therapeutic for ischemic stroke in C57BL/6J and CD1 mouse strains. PLoS One 12, e0183909, doi:10.1371/journal.pone.0183909 (2017). 28902897 PMC5597132

[14] Hancock A. M. & Frostig R. D. Hypertension prevents a sensory stimulation-based collateral therapeutic from protecting the cortex from impending ischemic stroke damage in a spontaneously hypersensitive rat model. PLoS One 13, e0206291, doi:10.1371/journal.pone.0206291 (2018). 30352082 PMC6198990

[15] Zhu J. Spatiotemporal dynamics of pial collateral blood flow following permanent middle cerebral artery occlusion in a rat model of sensory-based protection: a Doppler optical coherence tomography study. Neurophotonics 6, 045012, doi:10.1117/1.NPh.6.4.045012 (2019). 31824979 PMC6903432

[16] Wann E. G., Wodeyar A., Srinivasan R. & Frostig R. D. Rapid development of strong, persistent, spatiotemporally extensive cortical synchrony and underlying oscillations following acute MCA focal ischemia. Sci Rep 10, 21441, doi:10.1038/s41598-020-78179-4 (2020). 33293620 PMC7722868

[17] Rasheed W., Wodeyar A., Srinivasan R. & Frostig R. D. Sensory stimulation-based protection from impending stroke following MCA occlusion is correlated with desynchronization of widespread spontaneous local field potentials. Sci Rep 12, 1744, doi:10.1038/s41598-022-05604-1 (2022). 35110588 PMC8810838

[18] Bhatti M. S. & Frostig R. D. Astrocyte-neuron lactate shuttle plays a pivotal role in sensory-based neuroprotection in a rat model of permanent middle cerebral artery occlusion. Sci Rep 13, 12799, doi:10.1038/s41598-023-39574-9 (2023). 37550353 PMC10406860

[19] Burnett M. G. Electrical forepaw stimulation during reversible forebrain ischemia decreases infarct volume. Stroke 37, 1327–1331, doi:10.1161/01.STR.0000217305.82123.d8 (2006). 16556880

[20] Bandla A. Peripheral sensory stimulation is neuroprotective in a rat photothrombotic ischemic stroke model. Annu Int Conf IEEE Eng Med Biol Soc 2016, 6086–6089, doi:10.1109/EMBC.2016.7592117 (2016). 28269641

[21] Liao L. D. Rescue of cortical neurovascular functions during the hyperacute phase of ischemia by peripheral sensory stimulation. Neurobiol Dis 75, 53–63, doi:10.1016/j.nbd.2014.12.022 (2015). 25573087

[22] Pan H. C. Neurovascular function recovery after focal ischemic stroke by enhancing cerebral collateral circulation via peripheral stimulation-mediated interarterial anastomosis. Neurophotonics 4, 035003, doi:10.1117/1.NPh.4.3.035003 (2017). 28983488 PMC5621356

[23] Liao L. D. Improving neurovascular outcomes with bilateral forepaw stimulation in a rat photothrombotic ischemic stroke model. Neurophotonics 1, 011007, doi:10.1117/1.NPh.1.1.011007 (2014). 26157965 PMC4478786

[24] Peruzzotti-Jametti L. Safety and efficacy of transcranial direct current stimulation in acute experimental ischemic stroke. Stroke 44, 3166–3174, doi:10.1161/STROKEAHA.113.001687 (2013). 23982710

[25] Wu J. F. Efficacy of transcranial alternating current stimulation over bilateral mastoids (tACS(bm)) on enhancing recovery of subacute post-stroke patients. Top Stroke Rehabil 23, 420–429, doi:10.1080/10749357.2016.1175218 (2016). 27145292

[26] Liu Y. H. Integrated treatment modality of cathodal-transcranial direct current stimulation with peripheral sensory stimulation affords neuroprotection in a rat stroke model. Neurophotonics 4, 045002, doi:10.1117/1.NPh.4.4.045002 (2017). 29021986 PMC5627795

[27] Guo T. Pulsed Transcranial Ultrasound Stimulation Immediately After The Ischemic Brain Injury is Neuroprotective. IEEE Trans Biomed Eng 62, 2352–2357, doi:10.1109/TBME.2015.2427339 (2015). 25935023

[28] Bo B., Li Y., Li W., Wang Y. & Tong S. Optogenetic Excitation of Ipsilesional Sensorimotor Neurons is Protective in Acute Ischemic Stroke: A Laser Speckle Imaging Study. IEEE Trans Biomed Eng 66, 1372–1379, doi:10.1109/TBME.2018.2872965 (2019). 30281433

[29] Frostig R. D., Lieke E. E., Ts’o D. Y. & Grinvald A. Cortical functional architecture and local coupling between neuronal activity and the microcirculation revealed by in vivo high-resolution optical imaging of intrinsic signals. Proc Natl Acad Sci U S A 87, 6082–6086, doi:10.1073/pnas.87.16.6082 (1990). 2117272 PMC54476

[30] Ts’o D. Y., Frostig R. D., Lieke E. E. & Grinvald A. Functional organization of primate visual cortex revealed by high resolution optical imaging. Science 249, 417–420, doi:10.1126/science.2165630 (1990). 2165630

[31] Chen-Bee C. H., Agoncillo T., Xiong Y. & Frostig R. D. The triphasic intrinsic signal: implications for functional imaging. J Neurosci 27, 4572–4586, doi:10.1523/JNEUROSCI.0326-07.2007 (2007). 17460070 PMC6673004

[32] Bederson J. B. Evaluation of 2,3,5-triphenyltetrazolium chloride as a stain for detection and quantification of experimental cerebral infarction in rats. Stroke 17, 1304–1308, doi:10.1161/01.str.17.6.1304 (1986). 2433817

[33] Tamura A., Graham D. I., McCulloch J. & Teasdale G. M. Focal cerebral ischaemia in the rat: 1. Description of technique and early neuropathological consequences following middle cerebral artery occlusion. J Cereb Blood Flow Metab 1, 53–60, doi:10.1038/jcbfm.1981.6 (1981). 7328138

[34] Schneider C. A., Rasband W. S. & Eliceiri K. W. NIH Image to ImageJ: 25 years of image analysis. Nat Methods 9, 671–675, doi:10.1038/nmeth.2089 (2012). 22930834 PMC5554542

[35] Paxinos G. & Watson C. The rat brain in stereotaxic coordinates: hard cover edition. (Elsevier, 2006).

[36] Schummers J., Yu H. & Sur M. Tuned responses of astrocytes and their influence on hemodynamic signals in the visual cortex. Science 320, 1638–1643, doi:10.1126/science.1156120 (2008). 18566287

[37] Ferezou I., Bolea S. & Petersen C. C. Visualizing the cortical representation of whisker touch: voltage-sensitive dye imaging in freely moving mice. Neuron 50, 617–629, doi:10.1016/j.neuron.2006.03.043 (2006). 16701211

[38] Dembitskaya Y. Lactate supply overtakes glucose when neural computational and cognitive loads scale up. Proc Natl Acad Sci U S A 119, e2212004119, doi:10.1073/pnas.2212004119 (2022). 36375086 PMC9704697

[39] Li K. P. Noninvasive Brain Stimulation for Neurorehabilitation in Post-Stroke Patients. Brain Sci 13, doi:10.3390/brainsci13030451 (2023). PMC10046557 36979261

